# Examination of the Synthetic Control Method for Evaluating Health Policies with Multiple Treated Units

**DOI:** 10.1002/hec.3258

**Published:** 2015-10-07

**Authors:** Noémi Kreif, Richard Grieve, Dominik Hangartner, Alex James Turner, Silviya Nikolova, Matt Sutton

**Affiliations:** ^1^ London School of Hygiene and Tropical MedicineLondonUK; ^2^ London School of EconomicsLondonUK; ^3^ Manchester Centre for Health EconomicsUniversity of ManchesterManchesterUK; ^4^ University of LeedsLeedsUK

**Keywords:** synthetic control method, difference‐in‐differences, policy evaluation, pay‐for‐performance

## Abstract

This paper examines the synthetic control method in contrast to commonly used difference‐in‐differences (DiD) estimation, in the context of a re‐evaluation of a pay‐for‐performance (P4P) initiative, the Advancing Quality scheme. The synthetic control method aims to estimate treatment effects by constructing a weighted combination of control units, which represents what the treated group would have experienced in the absence of receiving the treatment. While DiD estimation assumes that the effects of unobserved confounders are constant over time, the synthetic control method allows for these effects to change over time, by re‐weighting the control group so that it has similar pre‐intervention characteristics to the treated group.

We extend the synthetic control approach to a setting of evaluation of a health policy where there are multiple treated units. We re‐analyse a recent study evaluating the effects of a hospital P4P scheme on risk‐adjusted hospital mortality. In contrast to the original DiD analysis, the synthetic control method reports that, for the incentivised conditions, the P4P scheme did not significantly reduce mortality and that there is a statistically significant increase in mortality for non‐incentivised conditions. This result was robust to alternative specifications of the synthetic control method. © 2015 The Authors. *Health Economics* published by John Wiley & Sons Ltd.

## 1 Introduction

In the absence of randomised controlled trials, evaluations of alternative health policies and public health interventions may use evidence from natural experiments (Craig *et al.,* 2012; Jones and Rice, 2011). Natural experiments attempt to exploit exogenous variation in the participation in the programme under evaluation, for example, across regions or time periods. Difference‐in‐differences (DiD) methods are often used to estimate treatment effects in these settings, by contrasting the change in outcomes pre‐ and post‐intervention, for the treatment and control groups. An attractive feature of DiD estimation is that it can remove the portion of confounding due to time‐invariant (fixed) differences between the comparison groups in unobserved covariates that predict the outcome of interest, assuming that the effects of these unobserved confounders do not change over time. DiD also assumes that any time effects (e.g. macro shocks) are common to the treatment groups under evaluation. The combination of these two assumptions is often referred to as the ‘parallel trends assumption’, which implies that without the intervention, outcomes for the treated and control groups would have followed parallel trajectories over time. DiD estimation is an increasingly common approach for evaluating health programmes (e.g. Currie *et al.,* 2014; Échevin and Fortin, 2014). As a recent review by Ryan *et al.* (2014) points out, while the theoretical properties of DiD estimation are well understood, a major concern is whether in practice the parallel trends assumption is plausible. The authors present simulation evidence that the choice of specification for the DiD estimation can have a major impact on the point estimates and estimated statistical significance of estimated policy effects and suggest that alternatives to DiD also warrant consideration.

The synthetic control method, pioneered by Abadie and Gardeazabal (2003) and Abadie *et al.* (2010) is an alternative approach for programme evaluation that relaxes the parallel trends assumption. Abadie *et al*. (2010) introduced the synthetic control method in the context of comparative case studies, where only one or a few units are subject to intervention, while a larger set of units remained untreated. The central idea behind the synthetic control method is that the outcomes from the control units are weighted so as to construct the counterfactual outcome for the treated unit, in the absence of the treatment. More precisely, a synthetic control unit is defined as the time‐invariant weighted average of available control units, which prior to the intervention have similar pre‐intervention characteristics and outcome trajectory to the treated unit. In contrast to the DiD method, the synthetic control method allows the effects of observed and unobserved predictors of the outcome to change over time, while assuming that pre‐intervention covariates have a linear relationship with outcomes post‐treatment.

The synthetic control method therefore may be useful for health policy evaluations when the validity of the parallel trends assumption is questionable. However, the data structures from such studies typically differ to previous comparative case studies that have applied the synthetic control method, where only one or a few units are subject to intervention (Abadie and Gardeazabal, 2003; Abadie *et al.*, 2010; Eren and Ozbeklik, 2011; Coffman and Noy, 2012; Hinrichs, 2012; Billmeier and Nannicini, 2013). The synthetic control method has recently been considered for settings with multiple treated units, but this research is currently unpublished, and it is unclear whether the methods proposed apply to evaluations of alternative health policies (Acemoglu *et al.,* 2013; Dube and Zipperer, 2013; Xu, 2015).

In a health context, the synthetic control approach has been considered for macro‐level interventions including the effects of wage compensation on the migration of health professionals (Okeke, 2009), the impact of universal coverage on health outcomes (Courtemanche and Zapata, 2014), and the impact of expanding public insurance eligibility on insurance take‐up (Lo, 2013). We are not aware of a previous study in a health context that critically examines the synthetic control approach as a contrast to DiD estimation.

In this context, policy makers often introduce new initiatives at the level of the health care provider, and so multiple units are treated. For example, hospital pay‐for‐performance (P4P) schemes, which link a portion of the provider's income to achieving quality targets, tend to be introduced for individual providers, in spite of scant evidence on their effectiveness and cost‐effectiveness (Meacock *et al.,* 2014; Emmert *et al.,* 2012; Lagarde *et al.,* 2013; Epstein, 2012; Eijkenaar, 2013). One of the most rigorous evaluations of a P4P programme was the assessment of Advancing Quality (AQ), which was introduced in the North West of England in 2008 (Sutton *et al.,* 2012). The evaluation applied a DiD approach, exploiting a quasi‐experimental design, in that the programme was introduced in all 24 hospitals in the North West region, while all 132 hospitals in the rest of England served as a control group. The study reported that for conditions incentivised by the programme, risk‐adjusted hospital mortality was reduced (Sutton *et al.,* 2012), and the scheme was cost‐effective (Meacock *et al.,* 2014). This significant improvement in patient outcomes was in direct contrast to findings from a similar US scheme, the Hospital Quality Incentive Demonstration (Ryan, 2009). Possible explanations for the positive effects of the AQ scheme included the relatively large bonus payments for hospitals in the AQ scheme (Epstein, 2012), the scheme's design that encouraged cooperation amongst providers but also, as acknowledged by the authors of the original evaluation, potential bias due to residual confounding (Sutton *et al.,* 2012). Specifically, the reduction in risk‐adjusted mortality that was reported may have been due to unobserved covariates whose values differed across regions and whose effects varied over the study period.

The aim of this paper is to critically examine the synthetic control method, in the context of evaluating health policies with multiple treated units such as hospitals. We re‐evaluate the AQ scheme using the synthetic control method and contrast the findings to the original DiD analysis. In order to ensure comparability to the original DiD evaluation, we use risk‐adjusted mortality that controls for observed, patient‐level compositional differences that vary over time. We propose a modification of the approach originally proposed by Abadie *et al*. (2010) in using the synthetic control method to construct an aggregate synthetic control unit, using hospital‐level data from the control regions. To represent uncertainty, we extend the placebo tests proposed by Abadie *et al*. (2010) to accommodate the data structure with multiple treated units.

The remainder of the paper is organised as follows. In Section 2 we introduce the motivating example and the original DiD analysis. Section 3 provides a general overview of the synthetic control method. Section 4 describes our proposed approaches to modifying the synthetic control method for multiple treated units, and Section 5 presents the results. Section 6 presents robustness checks, and Section 7 concludes and discusses the limitations of the study.

## 2 Motivating Example: Evaluation of the Advancing Quality Scheme

The AQ scheme was the first hospital‐based P4P programme in the UK, introduced in October 2008 for all hospitals in the North West Strategic Health Authority. The AQ scheme aimed to improve hospital performance on a number of clinical processes by rewarding hospitals for achieving quality targets. As the US Hospital Quality Incentive Demonstration scheme, the AQ was initially based on a ‘tournament’ system, in which bonuses were paid to the top performers. The programme required participating hospitals to collect and submit data on 28 quality measures of patient care across five clinical areas: pneumonia, heart failure, acute myocardial infarction, hip and knee surgery, and coronary artery bypass grafting. In the first year of the programme, bonuses equal to 2–4% of their revenue were paid to hospitals, which reported quality scores in the top two quartiles. For the next 6 months, bonuses were available also to those who reached a certain threshold and those who made top improvements from performance in the first year. Earned bonuses were allocated internally to the clinical teams whose performance had earned the bonus.

We re‐analyse the data from the published evaluation of the programme's effects on patient outcomes (Sutton *et al.,* 2012), which was also used as the basis of the cost‐effectiveness evaluation (Meacock *et al.,* 2014). The study considered patients who were admitted with the three emergency conditions that the AQ programme was incentivised to change the management of, acute myocardial infarction, heart failure, and pneumonia, but also investigated the programme's potential effects on six clinical conditions not incentivised by the scheme (acute renal failure, alcoholic liver disease, intracranial injury, paralytic ileus, and duodenal ulcer).

**Figure 1:**
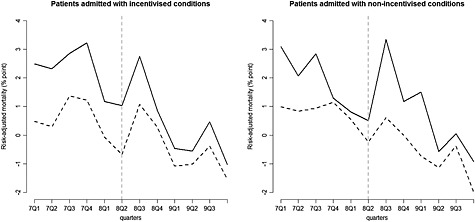
Risk‐adjusted 30‐day hospital mortality, in the North West of England (*n* = 24) (solid lines) vs. the rest of England (*n* = 122, sample constrained to create balanced panel; dashed lines), base case. The vertical dashed lines indicate the last pre‐intervention period

As in the original study, the outcome of interest is 30‐day risk‐adjusted hospital mortality, calculated using patient‐level data from the national Hospital Episode Statistics database (Health and Social Care Information Centre, 2014). Logistic regression, developed using the data from the pre‐intervention period only, was used to estimate a predicted risk score according to patient characteristics: age, sex, primary ICD‐10 diagnostic code, co‐morbidities, type, and location of admission. This predicted risk was aggregated to the hospital level, by quarter of the year, and subtracted from observed mortality rates. Figure 1 shows the trajectory of risk‐adjusted mortality in hospitals in the North West region and in the rest of England, six quarters before and after the start of the AQ.

The original DiD evaluation estimated the following panel data (fixed effects) regression model:

(1) $$Y_{\text{it}}=\delta_{t}+\lambda \mu_{i}+\alpha D_{\text{it}}+\varepsilon_{\text{it}}$$

where *i* is hospital and *t* is quarter of the year identifier. *Y*_*it*_ is risk‐adjusted mortality, *δ*_*t*_ are common time effects (time fixed effects), and *μ*_*i*_ are fixed hospital‐level unobserved variables (hospital fixed effects) with a time‐constant parameter *λ*, *D*_*it*_ is an indicator variable that takes the value of 1 for the hospitals in the North West after the programme start, and 0 otherwise, *α* corresponds to the effect of the treatment, and *ε*_*it*_ stands for idiosyncratic shocks with mean zero. The risk adjustment aimed to control for observed, time‐varying confounders measured at the patient level; hence, these variables are not further included in the hospital‐level regressions.

With DiD estimation, the parameter of interest is the ATT (Jones and Rice, 2011). Intuitively, the Average treatment effect on the treated (ATT) estimator contrasts the observed outcomes of the treated group to their counterfactual outcomes, after the intervention. 1In contrast, the ATE parameter captures the expected differences between counterfactual outcomes under treated and under control states, for everyone in the population.


This estimation approach can provide unbiased estimates of the ATT, if the effects of any hospital‐level factors, potentially leading to unobserved compositional differences between hospitals in the North West and the rest of England, remain constant over time and if the unobserved time effects are common across hospitals in treated and untreated hospitals.

The original DiD analysis reported that across the three incentivised conditions in the evaluation, the introduction of AQ led to a reduction in risk‐adjusted absolute 30‐day in‐hospital mortality by 0.9 percentage points [95% CI, −1.4 to −0.4], with a significant mortality reduction of 1.6 percentage points [95% CI, −2.4 to −0.8] for patients admitted with pneumonia. For patients admitted with the other two incentivised conditions, the estimated mortality reduction was not statistically significant. For the conditions considered in the evaluation that were not incentivised by AQ, the DiD analysis reported a non‐significant increase in mortality.

The differences in the pre‐intervention trajectories of risk‐adjusted mortality between the comparison groups (Figure 1) for both the incentivised but also the non‐incentivised conditions raise the concern that there may be unobserved covariates that differ between the comparison groups and whose effects on mortality change over time. Visual inspection of risk‐adjusted mortality in the periods before the introduction of the AQ scheme (Figure 1) raises concerns about whether the outcome trajectories for the North West region were parallel to those for the rest of England. Standard statistical tests could not reject the null hypothesis of parallel trends (Sutton *et al.,* 2012). However, the parallel trends assumption concerns treatment‐free potential outcomes in the pre‐treatment, but also in the post‐treatment, period and so this assumption is untestable, and the investigation of alternative methods that avoid this assumption are warranted.

## 3 Overview of the Synthetic Control Method

We first describe the synthetic control method for settings when only a single unit is exposed to treatment, following the notation of Abadie *et al*. (2010). Of the *J* + 1 units (regions), the first unit is exposed to a health policy, while the others remain unexposed and are referred to as the ‘donor pool’. Outcomes are observed for *T* periods, and the policy of interest starts in *T*_0_ + 1. The observed outcome vector of each region is $Y_{j}={\left(Y_{j1}\cdots Y_{jT_{0}}\cdots Y_{JT}\right)}^{\prime }$.

The observed outcome can be written as the sum of a treatment‐free potential outcome, *Y*_*jt*_^*N*^, and the effect of the treatment, *α*_*jt*_, such that

(2) $$Y_{jt}={Y_{jt}}^{N}+\alpha_{jt}D_{jt}$$

(3) $${Y_{jt}}^{N}=\delta_{t}+\;\lambda_{t}\mu_{j}+\;\theta_{t}Z_{j}+\varepsilon_{jt}$$

where *δ*_*t*_ is a time fixed effect, Z_*i*_ is a vector of time‐invariant measured predictors with time‐varying coefficient vector *θ*_*t*_, *μ*_*j*_ is a vector of time‐invariant unobserved predictor variables with time‐varying coefficients *λ*_*t*_, *D*_*jt*_ is an indicator variable that takes the value of 1 for the treated unit after *T*_0_, and is 0 otherwise, and *ε*_*jt*_ are unobserved transitory shocks with zero mean. Under the assumption that the relationship between the outcome and the predictors is linear, the synthetic control method generalises the DiD method by allowing the effects *λ*_*t*_ of the unobserved predictors *μ*_*j*_ to differ over time, while the DiD method constrains these effects to be constant (Equation 1).

Before the intervention, the treatment‐free potential outcome *Y*_*jt*_^*N*^ corresponds the observed outcome, for both the treated and the control regions. For periods after *T*_0_, the treatment‐free counterfactual for the treated region, *Y*_1*t*_^*N*^, is not observed. In order to estimate the treatment effect for the post‐intervention periods, the synthetic control method estimates the unobserved *Y*_1*t*_^*N*^ by creating a ‘synthetic control unit’, a weighted combination of potential controls that best approximates the relevant pre‐intervention characteristics of the treated region. Let the vector used for this weighting be *W* = (*w*_2_ ⋯ *w*_*J* + 1_)′, where w_*j*_ is the contribution of each control region to the synthetic control unit and the weights are constrained such that w_*j*_ ≥ 0 and *w*_2_ + ⋯ + *w*_*J* + 1_ = 1. The estimator of the counterfactual is constructed as the linear combination of the observed outcomes of the potential control regions: ${{\hat{Y}}^{N}}_{1t}=\sum_{j=2}^{J+1}w_{j}Y_{jt}$. The estimated treatment effect for the treated unit for each time period after *T*_0_ can then be obtained as ${\hat{\alpha }}_{1t}=Y_{1t}-\;{{\hat{Y}}^{N}}_{1t}$.

If the number of pre‐intervention periods is large relative to the scale of the idiosyncratic error (Abadie *et al*., 2010), an accurate prediction of pre‐intervention outcomes makes it more plausible that time‐varying responses to unobserved predictor variables are also similar between the treated and the synthetic control unit. 2The intuition is the following: The only way to reproduce the trajectories of the outcome variable over extended time periods is if the weighted controls are similar to the treated unit in both observed and unobserved predictors of the outcome variable, as well as in the effects of these variables on the outcome (Abadie, 2013). Formally, if the weighted value of the observed covariates and pre‐treatment outcomes for the control pool equals those of the treated region, $\sum_{j=2}^{J+1}w_{j}Z_{j}=Z_{1}$ and $\sum_{j=2}^{J+1}w_{j}Y_{jt}=Y_{1t},\;t=1,\ldots ,T_{o}$, and the outcome is a linear function of observed and unobserved potential confounders, then ${\hat{\alpha }}_{1t}$ is an approximately unbiased estimator of *α*_1*t*_.

The vector W* is chosen to minimise the discrepancy in the observed and unobserved confounders measured before the intervention, between the treated and the synthetic control region. The discrepancy is measured with the distance metric $\sqrt{{\left(X_{1}-X_{0}W\right)}^{\prime }V\left(X_{1}-X_{0}W\right)}$, where *X*_1_ is a *k* × 1 vector including *k* covariates and pre‐treatment outcomes for the treated region, while *X*_0_ is the corresponding *k* × *J* matrix of the control regions. *V* is a *k* × *k* positive definite and diagonal matrix, which assigns weights according to the relative importance of the covariates and the pre‐intervention outcomes. The choice of variables within *X*_0_ and *X*_1_ needs to be justified using substantive knowledge of the outcome process. While various choices are available for the method of selecting the weights in *V*, including the subjective assessment in their importance of predicting the outcome, Abadie *et al*. (2010) recommends jointly choosing *V* and *W* so that they minimise the root mean squared prediction error of the pre‐intervention outcomes.

To obtain the statistical significance of the estimated treatment effects, Abadie *et al*. (2010) propose a procedure akin to permutation tests (Edgington and Onghena, 2007). This involves iteratively re‐assigning treatment status for each control unit and, for this ‘placebo‐treated’ unit, re‐estimating the treatment effect by applying the synthetic control method, then comparing the estimated treatment effect to the distribution of placebo effects.

## 4 Extending the Synthetic Control Method for Multiple Treated Units

For evaluations where there is more than one treated unit, Abadie *et al*. (2010) suggests aggregating the treated units into a single treated unit, for example, a region. For the evaluation of the AQ, this would amount to aggregating the outcomes and covariates of the 24 hospitals in the North West to construct the treated region, as well as aggregating the outcomes of the control hospitals, to nine control regions. This would leave insufficient power to detect whether there was a statistically significant treatment effect. 3The minimum *p*‐value of rejecting the null hypothesis of no treatment effect, using a two‐sided test would be (1/9 × 2 = 0.22). Hence, we modify Abadie *et al*. (2010) approach and construct the synthetic control region by directly weighting the 132 control hospitals to match the average pre‐treatment outcomes of the hospitals in the treated region.

Let *i* be the hospital identifier and *t* the time period. *i* = 1 to *K*_1_ hospitals are treated, while the remaining *K*_1_ + 1 to *K*_1_ + *K*_2_ hospitals are controls. As before, the observed outcome of a hospital in each quarter can be written as *Y*_*it*_ = *Y*_*it*_^*N*^ + *α*_*it*_ *D*_*it*_. The aggregate outcome for the treated region can be defined as the previous expression weighted according to the relative frequency of the individual units (e.g. patients), so that

$${\bar{Y}}_{t}={{\bar{Y}}_{t}}^{N}+{\bar{\alpha }}_{t}D_{t}$$

where ${\bar{Y}}_{t}=\frac{\sum_{i=1}^{K_{1}}Y_{\text{it}}f_{\text{it}}}{\sum_{i=1}^{K_{1}}f_{\text{it}}},\;{{\bar{Y}}_{t}}^{N}=\frac{\sum_{i=1}^{K_{1}}{Y_{\text{it}}}^{N}f_{\text{it}}}{\sum_{i=1}^{K_{1}}f_{\text{it}}}$, ${\bar{\alpha }}_{t}=\frac{\sum_{i=1}^{K_{1}}\alpha_{\text{it}}f_{\text{it}}}{\sum_{i=1}^{K_{1}}f_{\text{it}}}\;,D_{t}$ is the treatment indicator, and *f*_*it*_ denotes patient numbers in each treated hospital and time period. As with DiD estimation, the synthetic control method with multiple treated units identifies the ATT parameter (Xu, 2015). We calculate the ATT by averaging the estimated treatments effects ${\bar{\alpha }}_{t}$ over the post‐treatment period, weighted by the time‐varying cumulative number of patients in the treated region.

We propose to use the synthetic control method to estimate the counterfactual outcome for the treated region, ${{\bar{Y}}_{t}}^{N}$, by re‐weighting the outcomes of the control hospitals:

$${{\hat{\bar{Y}}}_{t}}^{N}=\sum_{j=K_{1}+1}^{K_{1}+K_{2}}w_{j}Y_{jt}$$

The weight vector $W={\left(w_{K_{1}+1}\cdots w_{K_{1}+K_{2}}\right)}^{\prime }$ is selected to minimise the distance metric of the aggregated characteristics and pre‐treatment outcomes of the treated region and the synthetic control region (see Appendix 1 in the Supporting Information for further details).

We construct a test for the null hypothesis that the ATT for the treated region is zero, using a representation of the distribution of the ATT under the null. We obtain this distribution by exploiting potential permutations of hospitals into treated and control groups, accounting for a data structure where treatment is assigned at the level of the region. We build placebo‐treated regions by re‐sampling a set of hospitals from the donor pool only, without replacement, keeping the remaining controls in the donor pool. Then, we estimate the ATT using the synthetic control procedure outlined above. These two steps are repeated for a large number of independently re‐sampled placebo‐treated regions, allowing for repeatedly using control hospitals between placebo‐treated regions. We compare the distribution of the placebo ATTs to the observed ATT and calculate the *p*‐value of the two‐sided test as the proportion of placebo ATTs, which were at least as extreme in absolute value as the estimated ATT.

In the distance matrix we include hospital‐level covariates prior to the introduction of AQ (teaching hospital status, hospital quality indicator, the composition of admitted patients according to age, ethnicity, and predicted mortality) and pre‐intervention outcomes for each quarter. We replicate the original analysis in considering all the incentivised conditions aggregated, and then disaggregated by condition (pneumonia, acute myocardial infarction, heart failure). As in the original analysis, the non‐incentivised conditions were weighted according to patient numbers in each period, prior to the statistical analysis. We re‐construct the synthetic control region, for each analysis. In the placebo experiments, we create placebo regions that consist of the same number of hospitals as in the treated region (23 for the pneumonia condition and the non‐incentivised conditions, 24 for heart failure, myocardial infarction, and all incentivised conditions), and the number of placebo regions created is 100. We use the statistical software Stata throughout (StataCorp, 2011).

## 5 Results of the Main Analysis

**Figure 2:**
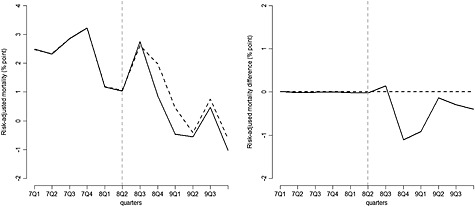
Left panel: Risk‐adjusted 30‐day hospital mortality, for patients admitted with incentivised conditions: the North West of England (solid line) vs. the synthetic North West (dashed line). Right panel: Gap in risk‐adjusted 30‐day hospital mortality for patients admitted with incentivised conditions: the North West of England vs. the synthetic North West. The vertical dashed lines indicate the last pre‐treatment period (base case)

We examine the synthetic control method in presenting step‐by‐step graphical results for the aggregate incentivised conditions (see Appendix 2 in the Supporting Information for results for each incentivised condition). Here, the results show that there is no difference in risk‐adjusted 30‐day hospital mortality, between the synthetic North West and the rest of England, prior to the introduction of the AQ scheme (up to 2008 quarter 3; Figure 2). Average hospital characteristics were also similar between the North West and the synthetic North West (Table 1). Synthetic control weights were non‐zero in each control region. No hospital contributed more than 25% of the synthetic control weights (see Appendix 3 in the Supporting Information). While the ‘gap’ between the predicted outcomes of the synthetic and the real North West before the programme started indicates the quality of the synthetic control region; the gap after the programme start can be attributed to the effect of AQ (Figure 2 right panel). Over the entire post‐intervention period, the estimated effect of AQ for the incentivised conditions is −0.45%, and the null hypothesis that AQ had no effect on risk‐adjusted mortality for the incentivised conditions cannot be rejected (*p* = 0.21), whereas for the DiD, the estimated ATT is −0.88%, and the null is rejected (*p* < 0.01; Table 2).

**Table 1:** Means of hospital characteristics and outcomes measured before the AQ programme, for all incentivised conditions

	North West		Rest of England (mean)
	Real (mean)	Synthetic (mean)	
Teaching or specialist hospital	16%	15%	21%
Age	72.3	72.1	73.0
White	84%	84%	79%
National regulator's quality rating	3.47	3.46	3.48
Predicted mortality	18%	18%	19%
Risk‐adjusted mortality 07Q1 (% point)	2.48	2.47	0.48
Risk‐adjusted mortality 07Q2 (% point)	2.31	2.32	0.30
Risk‐adjusted mortality 07Q3 (% point)	2.85	2.85	1.37
Risk‐adjusted mortality 07Q4 (% point)	3.22	3.22	1.22
Risk‐adjusted mortality 08Q1 (% point)	1.17	1.18	−0.08
Risk‐adjusted mortality 08Q2 (% point)	1.03	1.05	−0.67

Averages of hospital‐level variables, weighted for number of patients admitted. Risk‐adjusted mortality is a % point difference between observed and predicted mortality of hospitals, aggregated to the region level.

**Table 2:** Estimated ATTs: DiD vs. synthetic control method (base case)

	Standard DiD ATT (*p*‐value^a^)	Synthetic control ATT (*p*‐value^b^)
All incentivised	−0.88 (<0.01)	−0.45 (0.21)
Pneumonia	−1.62 (<0.01)	−0.43 (0.45)
Heart failure	−0.29 (0.52)	0.74 (0.18)
Acute myocardial infarction	−0.28 (0.44)	−0.49 (0.15)
All non‐incentivised conditions	0.39 (0.30)	0.90 (0.02)

After discarding 10 hospitals that did not contribute observations to each quarter, we apply both methods on a balanced panel of 24 treated and 122 potential control hospitals.

a *p*‐values based on standard errors from fixed effects model, clustered by hospital.

b *p*‐values calculated based on placebo tests.

**Figure 3:**
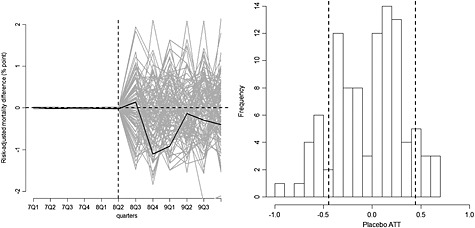
(a) Gap in risk‐adjusted 30‐day hospital mortality for patients admitted with incentivised conditions: the North West of England vs. the synthetic North West (black line) compared with the distribution of 100 placebo gaps (grey lines). The vertical dashed line indicates the last pre‐treatment period. (b) The distribution of estimated placebo ATTs. The dashed lines indicate the estimated ATT and −1*ATT (base case)

Figure 3 (left panel) shows the estimated gaps for the 100 placebo regions for the incentivised conditions, demonstrating a good pre‐intervention fit, and for the post‐intervention period, randomly scattered around zero. The right panel shows the empirical distribution of the placebo ATTs. The areas outside the two vertical lines show the proportion of the placebo ATTs that are more extreme than the estimated ATT of −0.45, translating to a *p*‐value of 0.21.

**Figure 4:**
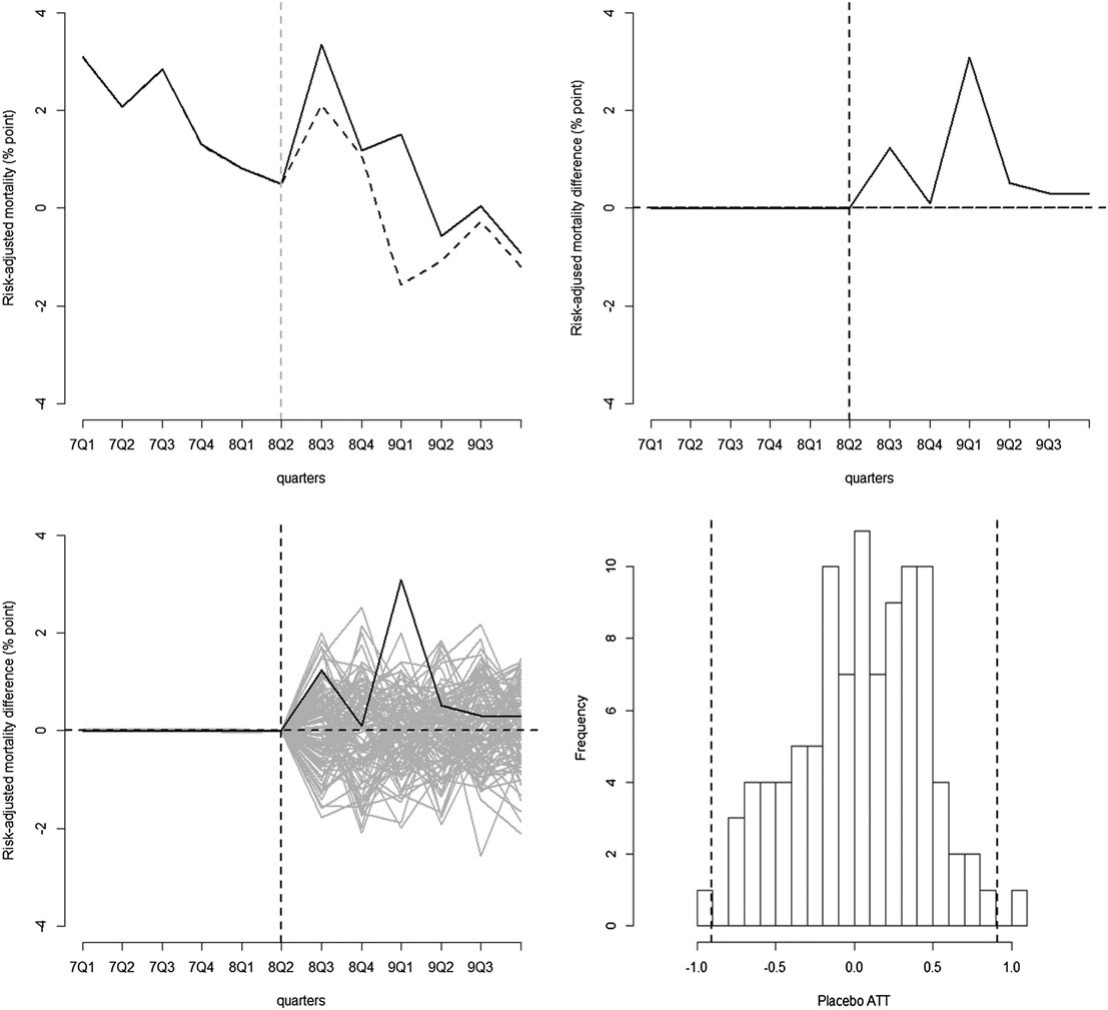
Graphical results of the synthetic control method: patients admitted with non‐incentivised conditions (base case)

The synthetic control method also reports a smaller ATT for pneumonia patients compared with the DiD analysis, and again the null hypothesis cannot be rejected (Table 2). For the heart failure and acute myocardial infarction patients, the synthetic control and DiD analyses both conclude that the null hypothesis cannot be rejected. For the non‐incentivised conditions, the synthetic control approach, unlike the DiD analysis, reports that the introduction of the AQ scheme lead to a large, significant (*p* < 0.01) increase in risk‐adjusted 30‐day hospital mortality (Figure 4).

## 6 Robustness Checks

### 6.1 Synthetic control method at the hospital level

We also consider an approach where we construct a synthetic control unit for each treated hospital. We estimate treatment effects at the hospital level and aggregate these to region level, using patient frequency weights of the treated hospitals. We also modify the placebo experiments to reflect the structure of the hospital‐level analysis (for details, see Appendix 4 in the Supporting Information). We find that the conclusions are consistent with those from the base case analysis (Table 3). The somewhat differing point estimates can be explained with the worse pre‐treatment fit obtained with the hospital‐level analysis (Appendix 5 in the Supporting Information). In order to correct for suboptimal pre‐treatment fit, we also combine the hospital‐level synthetic control analysis with DiD adjustment. After the synthetic control procedure, we create a new panel dataset including the treated hospitals and the synthetic control hospitals and apply DiD. 4In this robustness check, the estimated uncertainty is conditional on this reweighted data and does not take into account uncertainty in the process of creating the synthetic control hospitals. We find that the conclusions from the synthetic control method are robust for combination with DiD; however, the point estimates move closer to the results obtained from the aggregate analyses (Table 3).

**Table 3:** Estimated ATTs across methods: robustness checks

	(1) Synthetic control (base case)	(2) Synthetic control (synthetic control for each treated hospital)	(3) Synthetic control (synthetic control for each treated hospital) + DiD	(4) Standard DiD (base case)	(5) Matching + DiD
All incentivised	−0.45 (0.21)	−0.17 (0.55)	−0.27 (0.36)	−0.88 (0.00)	−0.74 (0.02)
Pneumonia	−0.43 (0.45)	−0.09 (0.95)	−0.24 (0.57)	−1.62 (0.00)	‐ 1.12 (0.02)
Heart failure	0.74 (0.18)	0.71 (0.13)	0.72 (0.16)	−0.29 (0.52)	0.51 (0.37)
Acute myocardial infarction	−0.49 (0.15)	−0.42 (0.23)	−0.72 (0.26)	−0.28 (0.44)	−0.12 (0.78)
Non‐incentivised	0.90 (0.02)	1.21 (0.01)	1.24 (<0.01)	0.39 (0.30)	0.86 (0.06)

*p*‐values in brackets. For (1) and (2), *p*‐values are based on placebo tests. For (3), (4), and (5), *p*‐values are based on standard errors from fixed effects regression, clustered by hospital.

### 6.2 Differences in differences combined with matching

We combine a multivariate matching method (Sekhon, 2011) with DiD, in order to reduce differences in observed confounders that might be associated with the outcome dynamics (Abadie, 2005) and increase the plausibility of the parallel trends assumption. We match on those same potential confounders (averaged over the pre‐treatment periods) and pre‐treatment outcomes that were included in the distance matrix of the synthetic control method. Matching resulted in good balance on all the predictors, while it was less successful in closely matching the pre‐treatment outcomes than the synthetic control method (Appendix 6 in the Supporting Information). We find that the treatment effects estimated for incentivised conditions overall and for pneumonia patients remain significant, but they attenuate in absolute value, compared with the unmatched DiD analysis. The estimated mortality difference for the non‐incentivised conditions increases from 0.39 (*p* = 0.30) to 0.86 (*p* = 0.06), which is close to the estimate obtained with the synthetic control approaches.

### 6.3 Leave‐one‐out robustness check for aggregate‐level synthetic control analysis

To examine whether the synthetic control results were driven by a few influential control hospitals, we also assess sensitivity of the estimated treatment effects to the iterative deletion of hospitals from the donor pool (Abadie *et al.,* 2015). Accounting for the regional structure of the data, we estimate nine new synthetic North West regions, in each of these analyses deleting one of the control regions. We find that the results are robust to the elimination of one control region a time (Appendix 7 in the Supporting Information).

## 7 Discussion

This paper examines the synthetic control method in the context of an evaluation of a high profile health policy change. Our empirical example, a re‐evaluation of a P4P scheme, has characteristics typical for the evaluation of health policies: there are multiple treated units, and the validity of the parallel trends assumption is questionable. We extend the synthetic control approach that Abadie *et al*. (2010) proposes, by re‐weighting disaggregated control units, in constructing a synthetic control region. We also present a procedure for inference, which accounts for a data structure with multiple treated units. This approach differs from previous applications of the synthetic control method, which predominantly estimate treatment effects for a single treated unit, such as a geographical region (Billmeier and Nannicini, 2013; Coffman and Noy, 2012; Eren and Ozbeklik, 2011; Okeke, 2009).

This paper extends the limited extant literature on the synthetic control method for multiple treated units. A working paper by Acemoglu *et al.* (2013) uses the synthetic control method to construct the treatment‐free potential outcome for each multiple treated unit and is similar to the approach we take in the sensitivity analysis, but weights the estimated unit‐level treatment effects according to the closeness of the synthetic control. Their inferential procedure is similar to the one developed here, in that they re‐sample placebo‐treated units from the control pool. Dube and Zipperer (2013) pool multiple estimates of treatment effects to generalise inference for a setting with multiple treated units and policies. Xu (2015) propose a generalisation for the synthetic control approach, for multiple treated units with a factor model that predicts counterfactual outcomes. Our approach is most closely related to the suggestion initially made by Abadie *et al*. (2010), to aggregate multiple treated units into a single treated unit. In preliminary simulation studies, we find the method reports relatively low levels of bias in similar settings to the AQ study.

We find that in our study, where the parallel trends assumption might be violated, the synthetic control method led to different conclusions about the estimated effectiveness of the programme, compared with the DiD approach. The major difference between the underlying assumptions of the two methods is that while the DiD approach assumes that unobserved differences have time‐invariant effects on the outcome, the synthetic control approach allows these effects to vary over time. While in this setting, standard statistical tests using pre‐intervention outcomes could not reject the parallel trend assumption, the concern remains that unobserved baseline variables, for example, the geographical sparseness of areas, differed between the comparison groups pre‐intervention. The concern for DiD is that the effects of such variables on risk‐adjusted hospital mortality may well have differed over the study time period, for example, because of improvements in practice over time for patients requiring emergency admission for conditions such as pneumonia.

In our empirical example of re‐evaluating the AQ scheme, the synthetic control method reported that for three of the conditions incentivised by the scheme, the programme did not lead to significant changes in risk‐adjusted 30‐day hospital mortality. This is in contrast with the initial evaluation that took the DiD approach described and reported that the AQ scheme reduced risk‐adjusted 30‐day hospital mortality (Sutton *et al.,* 2012).

The finding that a hospital P4P scheme did not improve health outcomes for conditions incentivised by the scheme is similar to those from a growing body of systematic reviews (Eijkenaar, 2013). A large evaluation of a similar P4P scheme in the USA, the premier Hospital Quality Incentive Demonstration, used the same incentivised conditions and process measures as the AQ but reported an improvement only in the incentivised process measures (Williams *et al.,* 2005), not the patient outcomes (Ryan, 2009). A follow‐up study of the long‐term effects of the AQ found that the previous DiD estimates of the positive effect of the policy on the incentivised conditions was not maintained, but rather that in the longer term risk‐adjusted mortality for the incentivised conditions fell significantly more in the rest of England than in the North West (Kristensen *et al.,* 2014).

Our re‐analysis found that across six non‐incentivised conditions, the introduction of AQ appeared to lead to a significant increase in risk‐adjusted mortality. The evaluation of another UK P4P scheme, the Quality and Outcomes Framework, found that the quality of care declined for aspects of care not incentivised by the programme (Doran *et al.,* 2011; Campbell *et al.,* 2009). Previous literature evaluating P4P schemes have reported harmful effects of extrinsic motivation for intrinsically motivated professionals (Deci *et al.,* 1999), as well as the possible ‘gaming’ of incentive schemes (Gravelle *et al.,* 2010; Woolhandler *et al.,* 2012). Evidence for detrimental effects of P4P has also been found in other areas of public policy, such as education (Dranove and Jin, 2010; Fryer, 2011). While our study adds to the literature raising concerns about the unintended consequences of P4P schemes, further research to investigate the reasons why P4P schemes might lead to worse health outcomes is clearly warranted.

Our proposed implementation of the synthetic control method aimed to ensure comparability of the estimates with the original DiD analysis. Prior to both the DiD and synthetic control estimation, we used the same patient‐level risk adjustment undertaken in the original analysis to adjust for observed patient‐level characteristics that changed over time. Both the DiD and the synthetic control analysis estimated the ATT weighted for patient numbers, over post‐treatment periods. Both implementations of the synthetic control method used hospital‐level data but aggregated to the level of the North West at different stages of the analysis. While the base case analysis directly reweighted control hospitals to create the synthetic North West, the sensitivity analysis created synthetic control hospitals, and aggregated these to the region level. We found that the point estimates and their statistical significance were robust to the choice of approach.

Methodological guidance on DiD estimation encourages studies to recognise the clustering of observations within higher level units (Bertrand *et al.,* 2002; Cameron *et al.,* 2011; Brewer *et al.,* 2013). In the evaluation of the AQ, there were potentially two cross‐sectional levels of clustering (admissions within hospitals within regions) and a time dimension (12 quarters). While there were insufficient observations to apply widely used methods (e.g. cluster robust standard errors) for handling clustering at the regional level (only one treated region), we did allow for correlated errors within hospitals. The approach taken to allowing for the clustering within hospitals is consistent with how the permutation test was implemented in the synthetic control analysis. Here, each placebo region was constructed by re‐sampling hospitals from across the whole control pool of hospitals, rather than re‐sampling hospitals within each specific region, i.e. again respecting the clustering within hospital but not within region.

In a sensitivity analysis in the DiD estimation, we implemented a two‐way (by hospital and quarter) cluster robust variance–covariance matrix (Schaffer, 2012), but this led to similar results to the base case.

The intuition behind the synthetic control method is similar to that for a matching approach that aims to select an appropriate control group, by balancing the pre‐treatment characteristics of the comparison groups. In contrast to the synthetic control approach, the combination of matching and DiD still requires the parallel trends assumption, but after conditioning on a set of observed covariates (Blundell and Dias, 2009). In the sensitivity analysis, we constructed a control group where pre‐treatment covariates and levels of pre‐treatment outcomes were closely matched to treated hospitals. Matching changed the results from the original DiD estimates in the same direction as the synthetic control method. This offers further support for concerns that the main assumption underlying the DiD approach may not be plausible in settings where the differences between the comparison groups in pre‐intervention outcomes are relatively large (Abadie, 2005).

This paper has several limitations. First, both the synthetic control and the standard DiD approaches assumed that unobserved covariates, and the P4P scheme, had a linear additive effect on risk‐adjusted mortality. However, if the characteristics of treatment and control units are similar, even when the true data‐generating processes are non‐linear, linear models can still provide a good approximation. The ‘changes in changes’ model proposed by Athey and Imbens (2006) offers an alternative extension of the DiD approach, which relaxes the linearity assumption.

A second assumption pertaining to all the methods presented is that following the introduction of AQ, there were no compositional changes in the patients admitted to the hospitals, nor other differential innovations to the local health care system. Both the synthetic control and the DiD approaches assume that risk adjustment at the patient level adjusts for compositional differences, which change over time, before AQ was introduced.

Third, a specific concern for the synthetic control method is that the number of pre‐treatment periods may be insufficient and that the fit of the pre‐treatment outcomes might be due to chance, leaving residual differences in unobserved factors that have changing effects over time. Furthermore, with a large number of potential controls available, several weighted combinations of the control units may create equally good predictions of the pre‐treatment outcomes. The scale of transitory shocks can be larger at the disaggregated level of hospitals than at the aggregate level, in which case more time periods would be desirable to reduce potential bias in the estimated treatment effects. The similar conclusions reached by the different implementations of the synthetic control method suggest that the reported results are robust to the specific choice of weights assigned to the synthetic controls.

The placebo experiments provide a further assessment for the ability of the synthetic control units to reproduce the treatment‐free counterfactual. We find that the trajectories of the placebo outcomes are distributed symmetrically around zero for the incentivised and non‐incentivised conditions, indicating that the synthetic control method would be expected to estimate a zero effect when the true effect is zero. The statistically significant result reported for the non‐incentivised conditions on the other hand demonstrates the ability of the synthetic control method to detect non‐zero treatment effects, for this dataset.

There is currently little guidance on the practical implementation of the synthetic control approach, for example, regarding variable selection in the distance matrix. We included predictors in the distance matrix based on prior knowledge on predictors of the outcome, risk‐adjusted mortality. Recent work has proposed a criterion based on the mean squared prediction error of the post‐intervention outcomes as a criterion for covariate selection (Dube and Zipperer, 2013). When assessing alternative specifications of the distance matrix, we found that the estimated effects changed only slightly, without altering the conclusions. In addition to the *p*‐values reported, confidence sets can be calculated by inverting a rank statistic (Hodges and Lehmann, 1963), but before this procedure can be applied to more general settings with treatment effect heterogeneity (Rosenbaum, 2003), further research is required.

Further research is required to investigate the relative merits of the synthetic control approach compared with DiD methods, across a wide range of settings. Simulation studies are now warranted to report the relative performance of the methods, specifically according to alternative lengths of pre‐intervention time periods, the scale of the transitory shocks, and the potential non‐linearity of the data‐generating model.

## Supporting information

Supporting info item
