# Supplementary material for: Examination of the Synthetic Control Method for Evaluating Health Policies with Multiple Treated Units
Source: Health Econ. 2015 Oct 7;25(12):1514–28. doi: 10.1002/hec.3258 (PMC5111584; doi:10.1002/hec.3258)
Supplement: Supplementary file 1 — Supporting info item [file HEC-25-1514-s001.docx]

**Appendix 1 Further details on the synthetic control method for multiple treated units (base case analysis)**

Below we provide further details on the proposed implementation of the synthetic control method for multiple treated units. Choosing the vector of synthetic control weights, $W$, involves minimising the distance metric $\sqrt{\left( X_{1}-X_{0}W \right)^{'}V_{i}\left( X_{1}-X_{0}W \right)}$ where $X_{1}$ is now the vector of the aggregated characteristics of the treated region, and $X_{0}$ is the matrix of pre-treatment characteristics of the control hospitals**.**  The algorithm minimises the root mean squared error (RMSE) of the pre-intervention outcomes of the aggregate treated region. This procedure results in a weight vector $W=(w_{K_{1}+1}\ldots\ldots.w_{K_{1}+K_{2}})'$, the elements of which sum to 1. Using this weight vector, the counterfactual outcome vector is estimated as ${\hat{\bar{Y}_{t}}}^{N}=\sum_{j=K_{1}+1}^{K_{1}+K_{2}} w_{j}Y_{jt}$.

This approach assumes that the synthetic control weights implicitly incorporate the $f_{it}$ weights. The conditions which are necessary for ${\hat{\bar{\alpha}}}_{t}$ to be unbiased can be now written as:

$\sum_{K_{1}+1}^{{K_{1}+K}_{2}} w_{j}Z_{j}=\frac{\sum_{i=1}^{K_{1}} Z_{i}f_{i}}{\sum_{i=1}^{K_{1}} f_{i}}$ and

$\sum_{K_{1}+1}^{{K_{1}+K}_{2}} w_{j}Y_{jt}=\frac{\sum_{i=1}^{K_{1}} Y_{it}f_{it}}{\sum_{i=1}^{K_{1}} f_{it}}$,${t=1,\ldots,T}_{o}$ ,

requiring that the aggregate pre-treatment covariates and pre-treatment outcomes can be approximated with the linear combination of those for the control hospitals.

**Appendix 2: Graphical results for the base case synthetic control analysis, by incentivised conditions.**

| 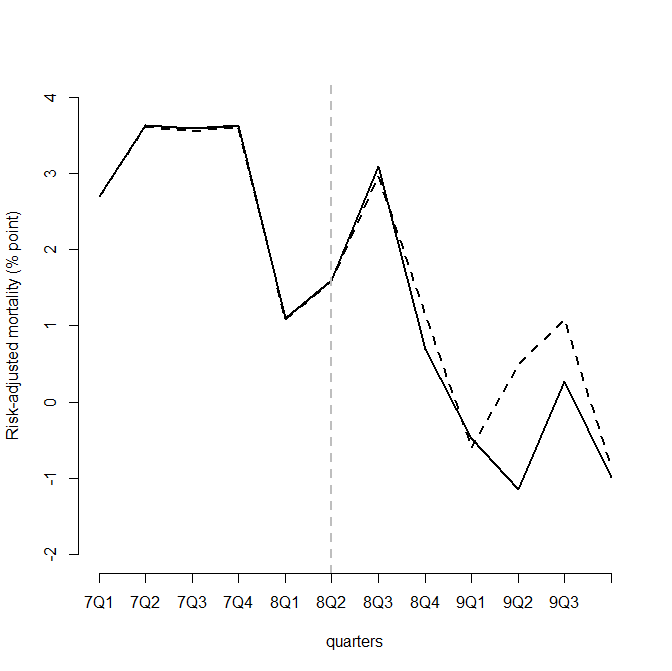 | 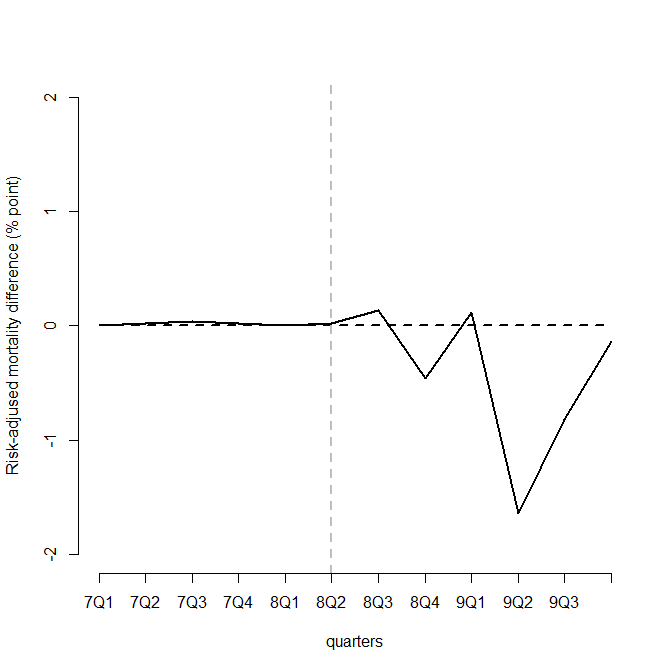 |
| --- | --- |
| 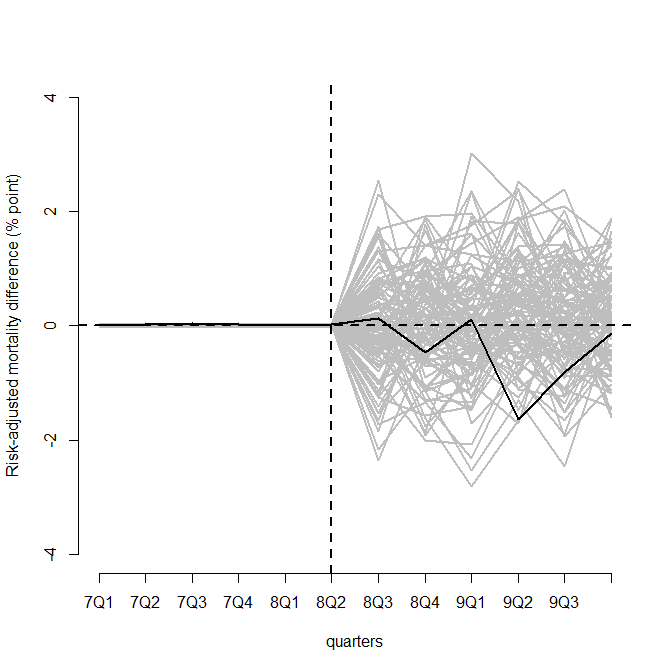 | 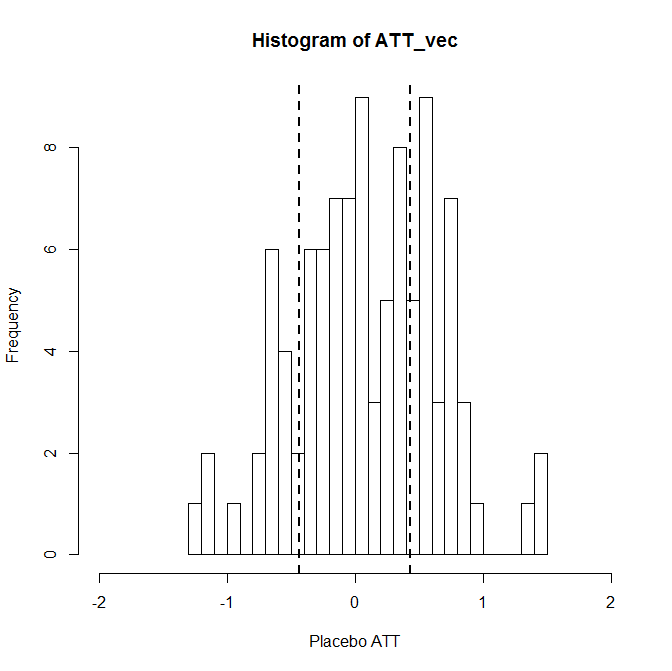 |

Figure 1 Graphical results of the synthetic control method (base case analysis): patients admitted with pneumonia

| 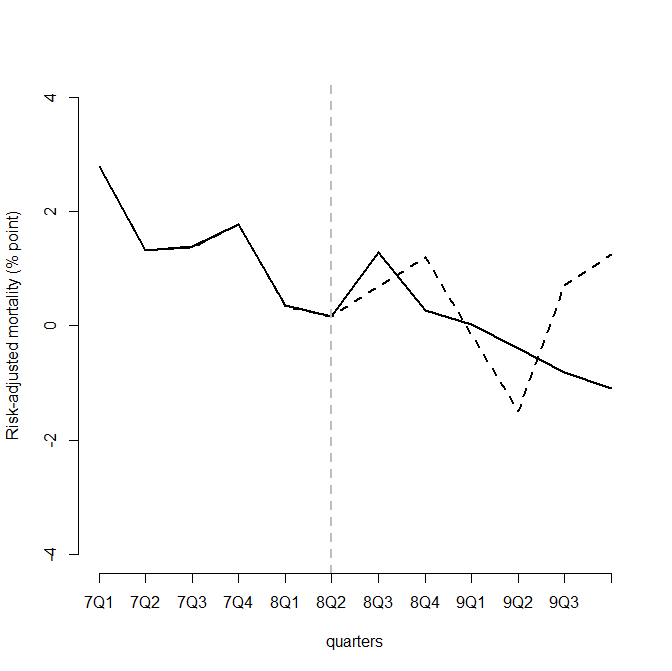 | 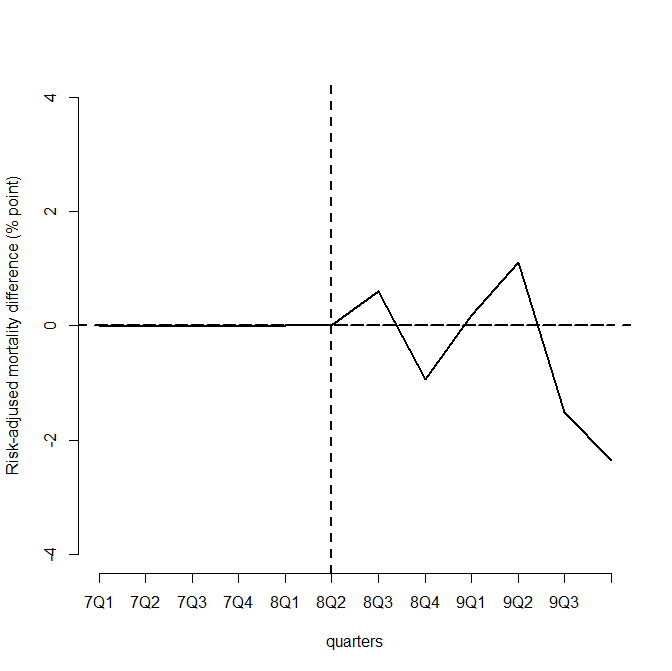 |
| --- | --- |
| 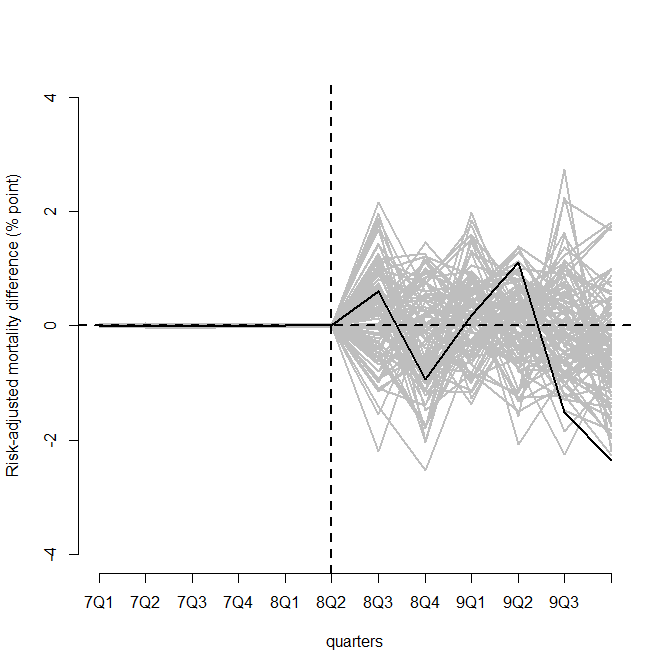 | 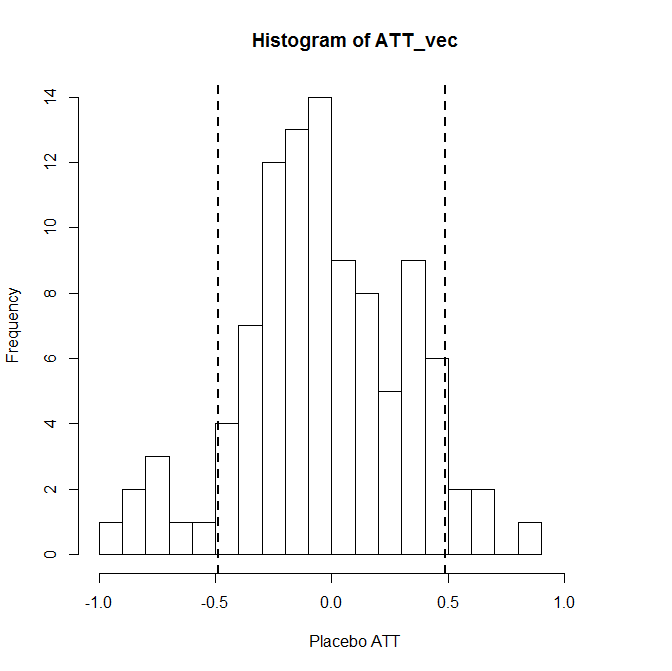 |

Figure 2 Graphical results of the synthetic control method (base case analysis): patients admitted with myocardial infarction

| 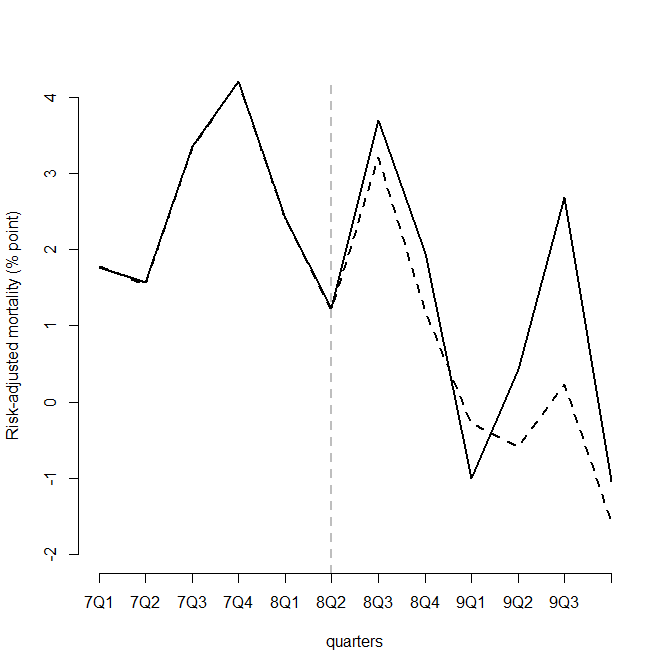 | 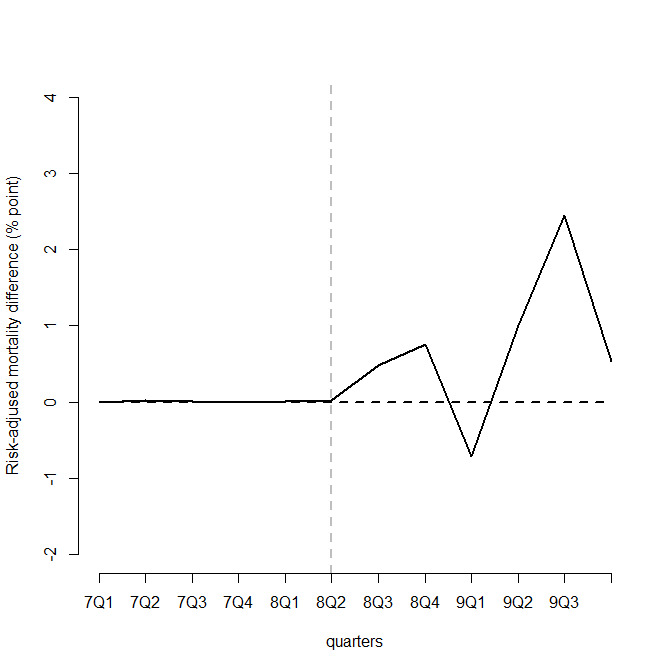 |
| --- | --- |
| 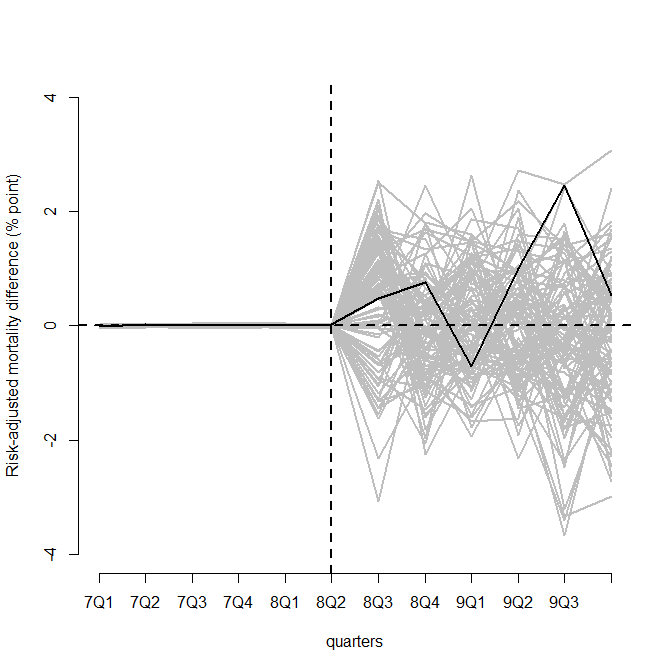 | 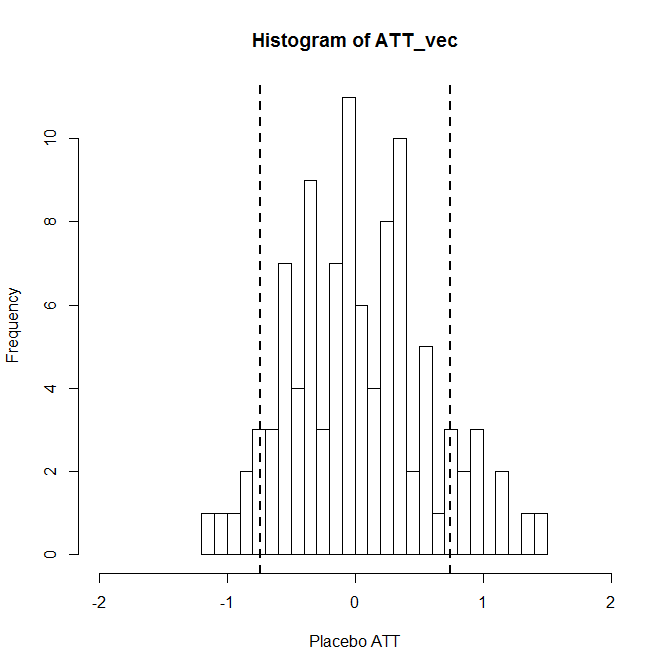 |

Figure 3 Graphical results of the synthetic control method (base case analysis): patients admitted with heart failure

**Appendix 3: Distribution of synthetic control weights (base case)**

Table 1 Distribution of synthetic control weights.

|    1. All incentivised conditions |    1. Non-incentivised conditions |
| --- | --- |
|    1. Pneumonia |    1. Heart failure |
|    1. Acute myocardial infarction |  |

**Notes:** Control hospitals are ordered on the horizontal axis by hospital identifier.

**Appendix 4: Sensitivity analysis: constructing synthetic control hospitals for each treated hospital**

Here we explore the use of the synthetic control procedure to construct a synthetic control unit for each treated hospital. This involves minimising the distance metric $\sqrt{\left( X_{1i}-X_{0}W_{i} \right)^{'}V_{i}\left( X_{1i}-X_{0}W_{i} \right)}$ for each treated hospital, where $X_{1i}$ is now the vector of pre-treatment characteristic of the treated hospital *i*, and $X_{0}$ is a matrix of pre-treatment characteristics of the control hospitals**.** This procedure results in a weight vector, $W_{i}=(w_{{iK}_{1}+1}\ldots\ldots.w_{{iK}_{1}+K_{2}})'$, and counterfactual outcome vector is $\hat{Y}_{it}^{N}=\sum_{j=K_{1}+1}^{K_{1}+K_{2}} w_{ij}Y_{jt}$ for each $i$ treated hospital. The treatment effect for each treated hospital can be estimated as
 $\hat{\alpha}_{\mathrm{it}}=Y_{\mathrm{it}}-{\hat{Y}^{N}}_{1t}$.

We then construct the observed and counterfactual trajectories of the average risk-adjusted mortality rate for the North West, using patient numbers as frequency weights. We create the average risk-adjusted mortality rate for the North West as the weighted average of the hospital level outcomes as $\bar{Y}_{1t}=\sum_{i=1}^{K_{1}} f_{it}Y_{it}/\sum_{i=1}^{K_{1}} f_{it}$. Similarly, we use the frequency weights to create the average predicted outcome for the synthetic North West, ${{\hat{\bar{Y}}}^{N}}_{1t}=\sum_{i=1}^{K_{1}} f_{it}\hat{Y}_{\mathrm{it}}/\sum_{i=1}^{K_{1}} f_{it}$. The treatment effect for each time period after $T_{0}$ can therefore be calculated as ${\hat{\bar{\alpha}}}_{1t}=\bar{Y}_{1t}-{{\hat{\bar{Y}}}^{N}}_{1t}$, which is equivalent to taking a weighted average of the hospital-level treatment effects,
 ${\hat{\bar{\alpha}}}_{t}={\sum_{i=1}^{K_{1}} f_{it}Y_{it}\hat{\alpha}}_{it}$ /$\sum_{i=1}^{K_{1}} f_{it}$.

The placebo-experiments reflect the structure of the hospital level-analysis. Here, each control hospital is assigned to placebo treatment. Hence, for each control hospital, a synthetic control hospital is constructed, using the donor pool of all other controls. Then, placebo treated regions are built, by randomly resampling a set of placebo treated hospitals for a large number of replications (here, 100 times), together with their synthetic control counterparts. For each of these placebo treated regions, the ATT is estimated, and the distribution of these ATTs is taken to represent the distribution under the null hypothesis of no treatment effect. P-values are again calculated as the proportion of placebo ATTs which were at least as extreme in absolute value as the estimated ATT.

**Appendix 5 - Graphical results for the sensitivity analysis - synthetic control hospitals constructed for each treated hospital**

| 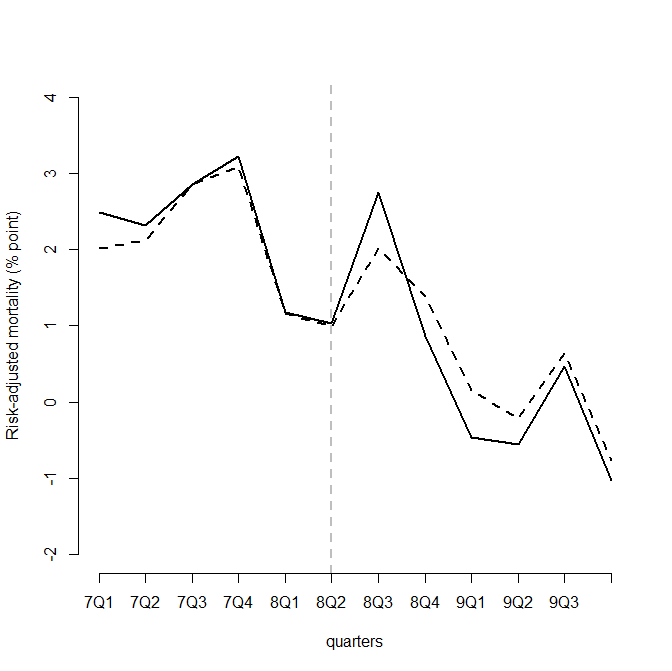 | 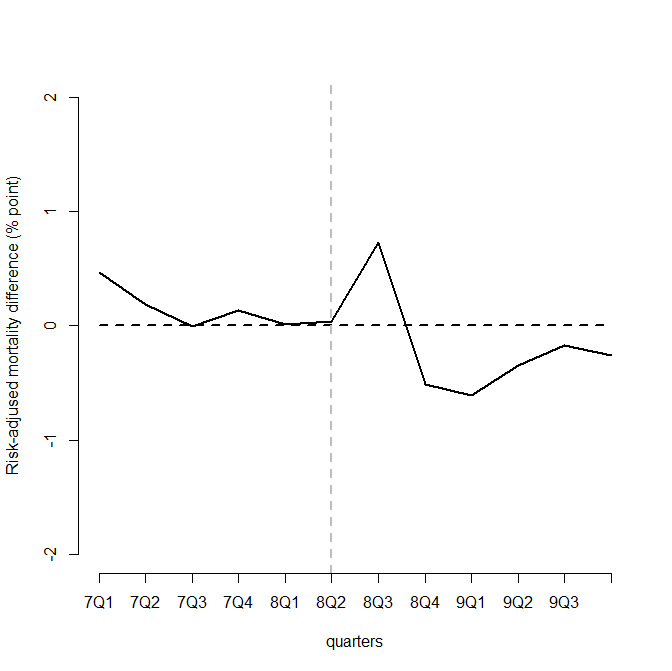 |
| --- | --- |
| 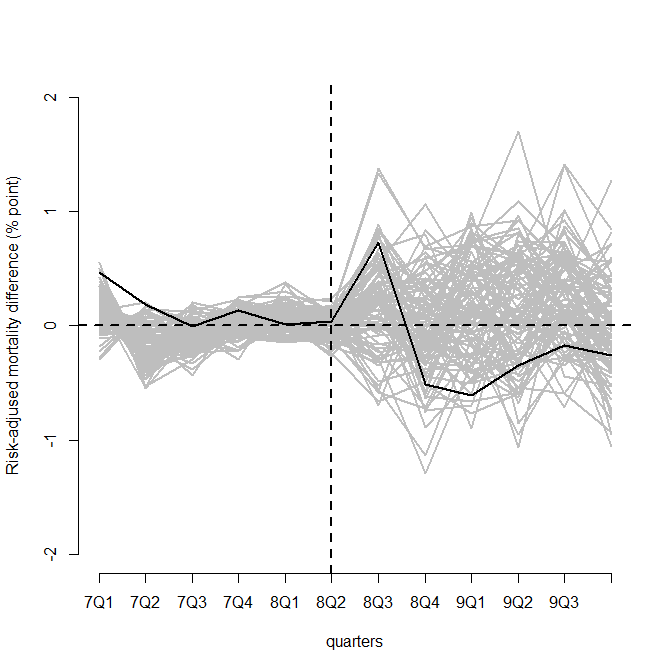 | 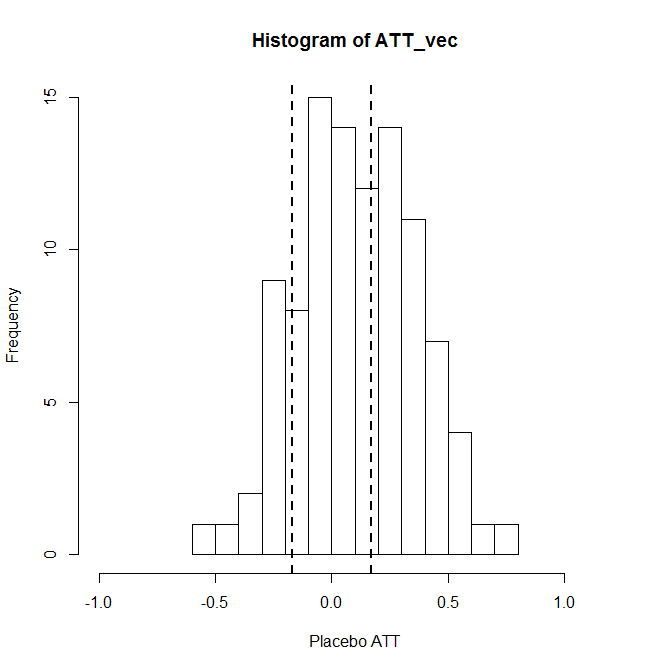 |

Figure 1 Graphical results of the synthetic control method (sensitivity analysis) - patients admitted with all incentivised conditions

| 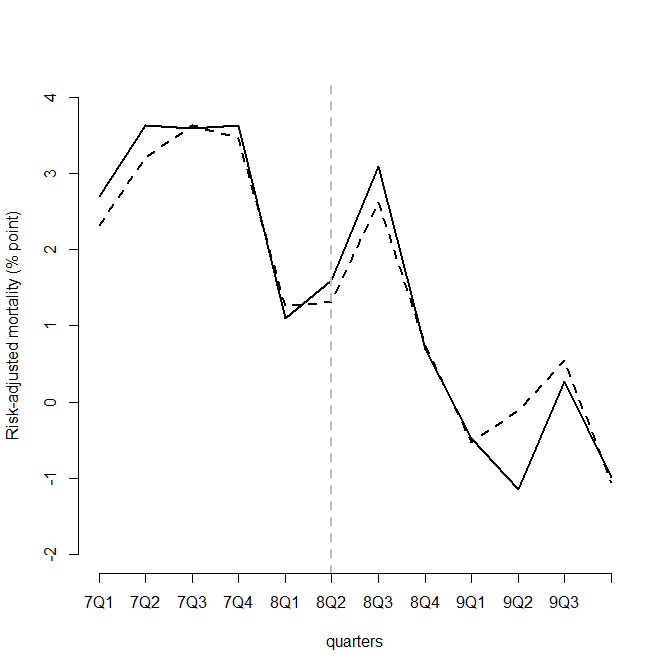 | 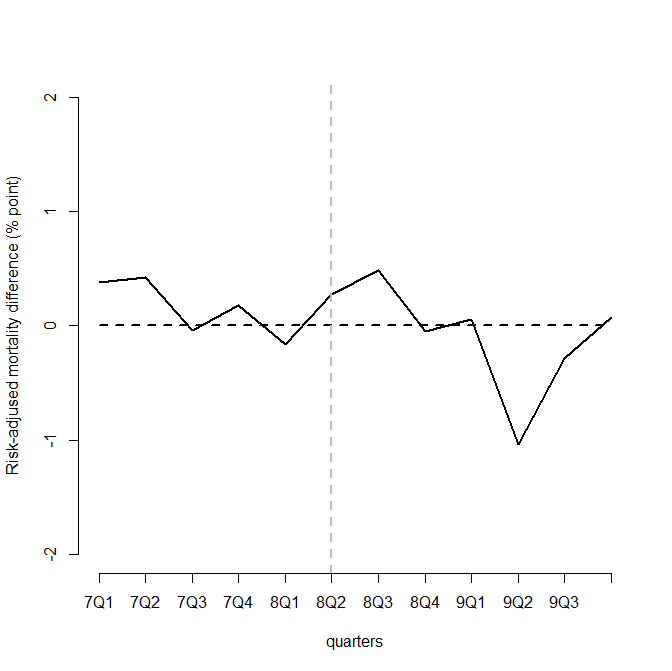 |
| --- | --- |
| 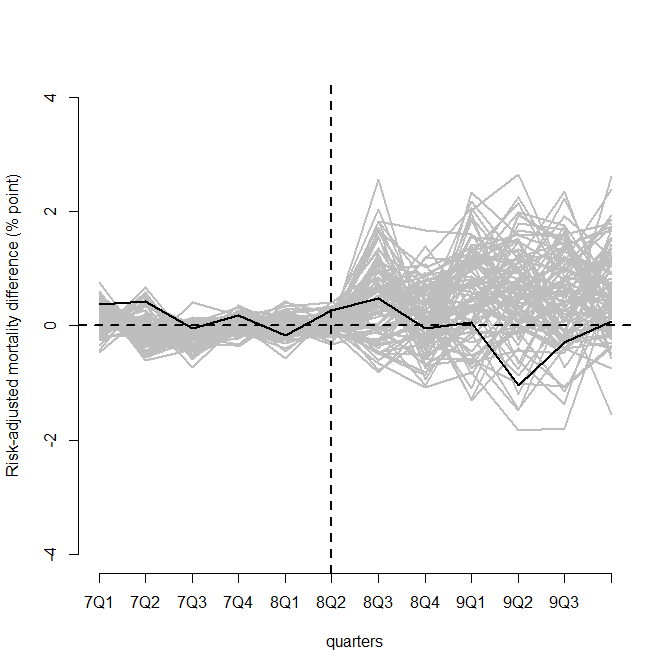 | 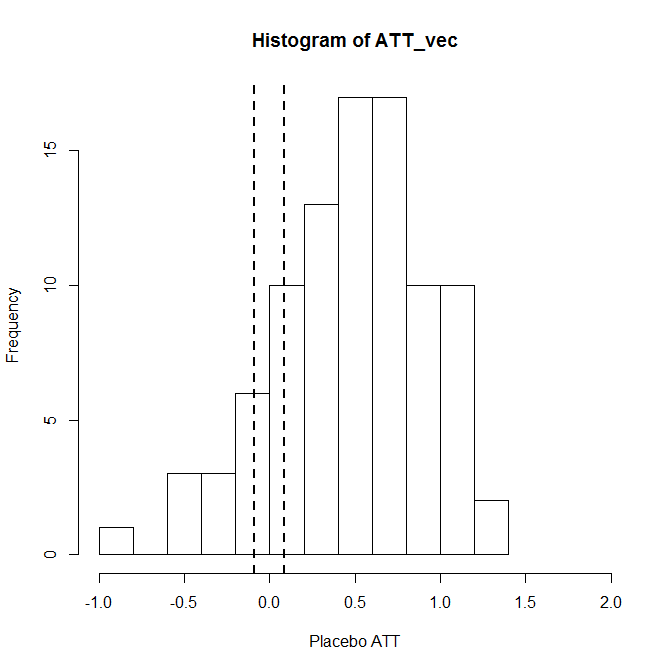 |

Figure 2 Graphical results of the synthetic control method (sensitivity analysis) - patients admitted with pneumonia

| 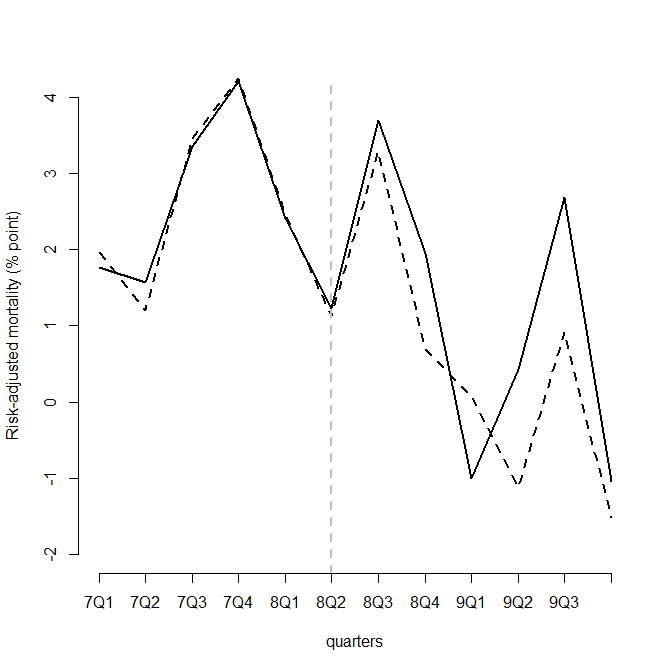 | 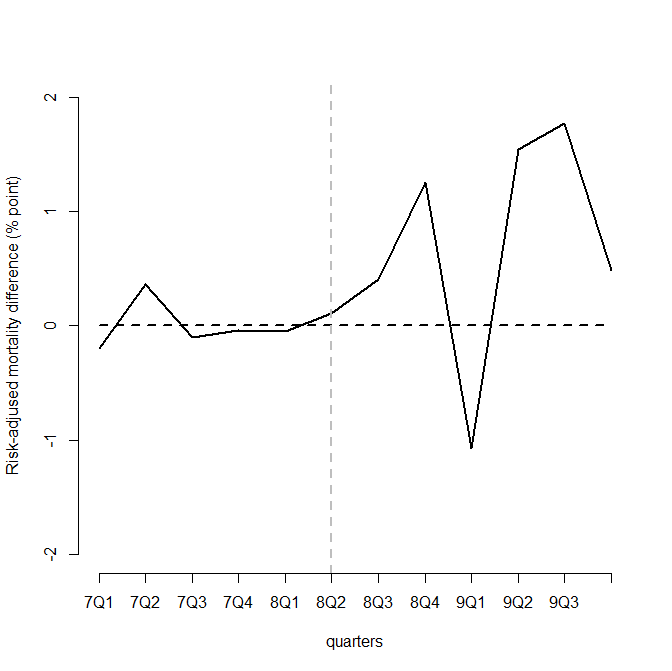 |
| --- | --- |
| 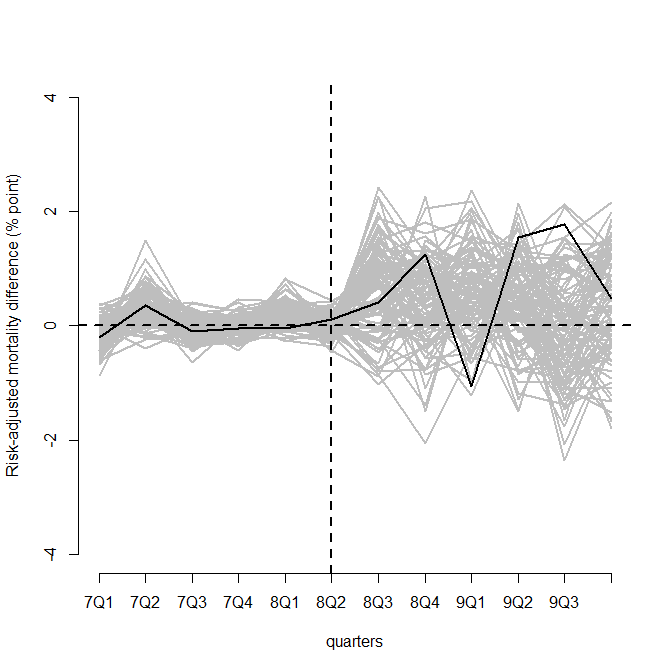 | 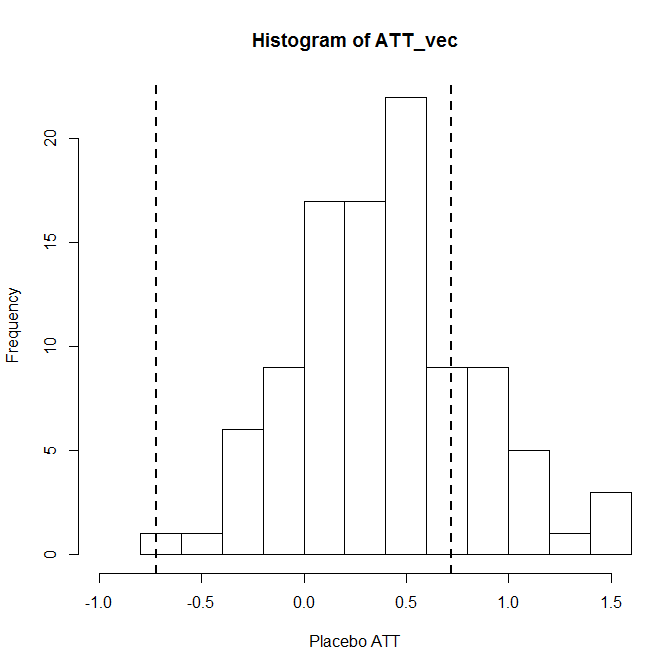 |

Figure 3 Graphical results of the synthetic control method (sensitivity analysis) - patients admitted with heart failure

| 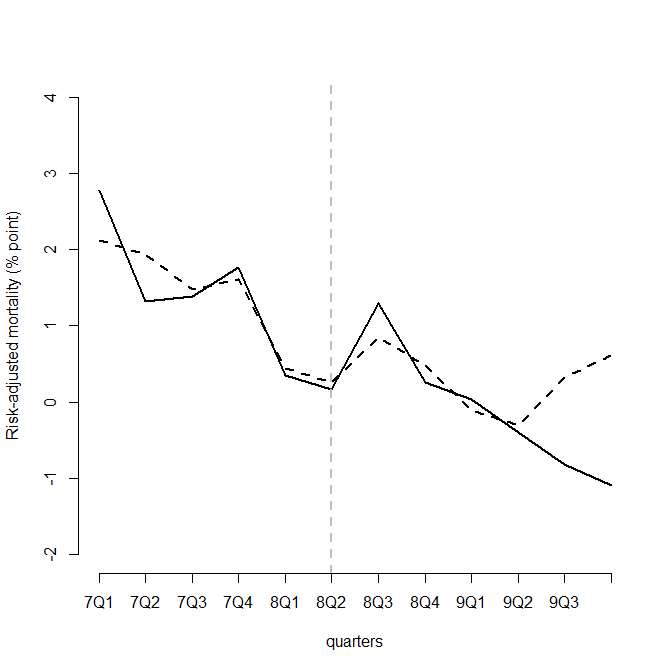 | 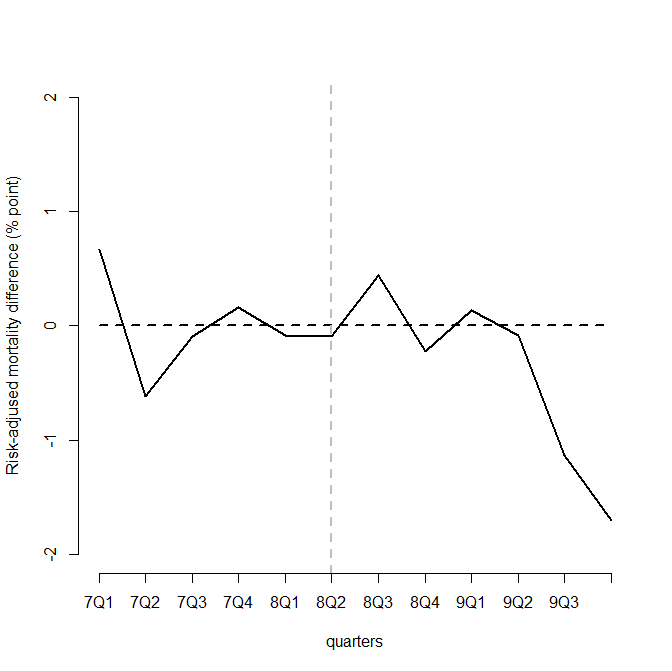 |
| --- | --- |
| 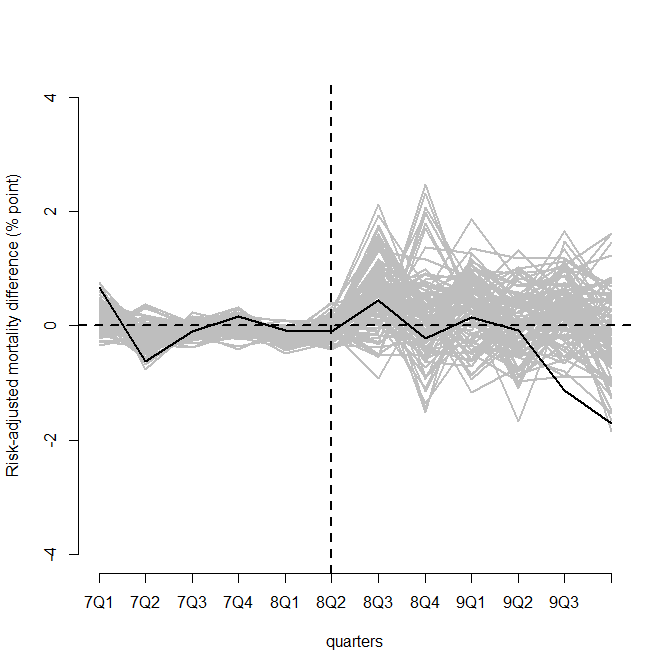 | 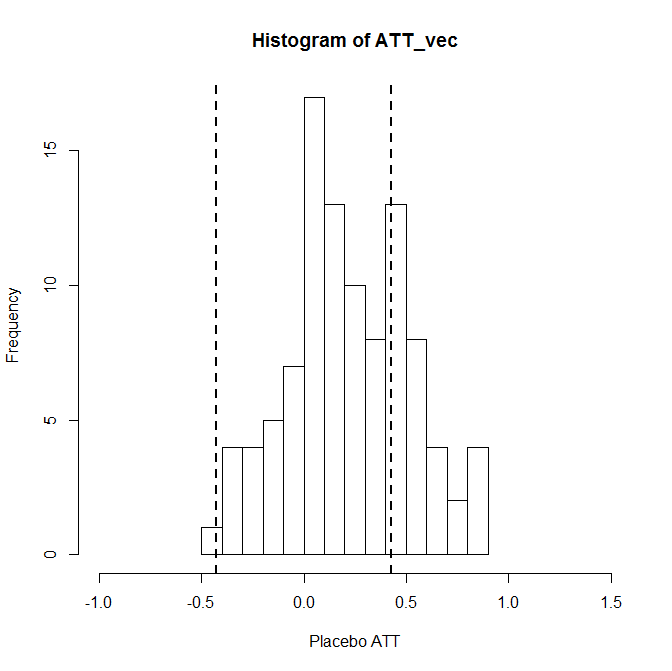 |

Figure 4 Graphical results of the synthetic control method (sensitivity analysis): patients admitted with acute myocardial infarction

**Appendix 6**

**Table 1 Balance of covariates and pre-treatment outcomes, after multivariate matching**

|  | **All incentivised conditions** | | **Pneumonia** | | **Heart failure** | | **Myocardial infarction** | | **Non-incentivised conditions** | |
| --- | --- | --- | --- | --- | --- | --- | --- | --- | --- | --- |
|  | **Mean, North West (n=24)** | **Mean, Rest of England (matched)**  **(n=24)** | **Mean, North West**  **(n=23)** | **Mean, Rest of England (matched)**  **(n=23)** | **Mean, North West**  **(n=24)** | **Mean, Rest of England (matched)**  **(n=24)** | **Mean, North West (n=24)** | **Mean, Rest of England (matched)**  **(n=24)** | **Mean, North West (n=23)** | **Mean, Rest of England (matched)**  **(n=23)** |
| Teaching/ specialist hospital (%) | 15.52 | 17.45 | 17.50 | 23.02 | 16.38 | 22.27 | 12.07 | 30.82 | 0.14 | 32.04 |
| Age | 72.28 | 73.14 | 71.79 | 72.51 | 76.30 | 76.47 | 70.15 | 70.35 | 61.83 | 62.63 |
| White (%) | 84.23 | 83.67 | 86.04 | 84.93 | 84.49 | 83.92 | 81.47 | 84.04 | 84.53 | 83.36 |
| New national targets score | 3.47 | 3.59 | 3.45 | 3.34 | 3.46 | 3.59 | 3.50 | 3.42 | 3.48 | 3.38 |
| Predicted mortality (%) | 18.43 | 18.72 | 25.29 | 26.17 | 15.44 | 15.38 | 11.10 | 10.98 | 11.53 | 11.17 |
| Risk-adjusted mortality 07Q1 (% point) | 2.48 | 1.95 | 2.69 | 2.06 | 1.77 | 1.36 | 2.78 | 1.55 | 3.09 | 3.00 |
| Risk-adjusted mortality 07Q2 (% point) | 2.31 | 1.92 | 3.63 | 3.29 | 1.57 | 1.38 | 1.32 | 0.09 | 2.06 | 1.78 |
| Risk-adjusted mortality 07Q3 (% point) | 2.84 | 2.55 | 3.58 | 3.31 | 3.35 | 2.25 | 1.39 | 1.21 | 2.84 | 1.26 |
| Risk-adjusted mortality 07Q4 (% point) | 3.22 | 2.49 | 3.62 | 3.19 | 4.20 | 2.74 | 1.77 | 0.82 | 1.29 | 1.38 |
| Risk-adjusted mortality 08Q1 (% point) | 1.17 | 1.11 | 1.09 | 1.27 | 2.41 | 1.18 | 0.35 | 0.26 | 0.80 | 0.85 |
| Risk-adjusted mortality 08Q2 (% point) | 1.03 | 0.54 | 1.59 | 0.91 | 1.22 | -0.18 | 0.16 | -0.34 | 0.50 | 0.59 |

**Note**: covariate means weighted by patient numbers in each hospital and quarter.


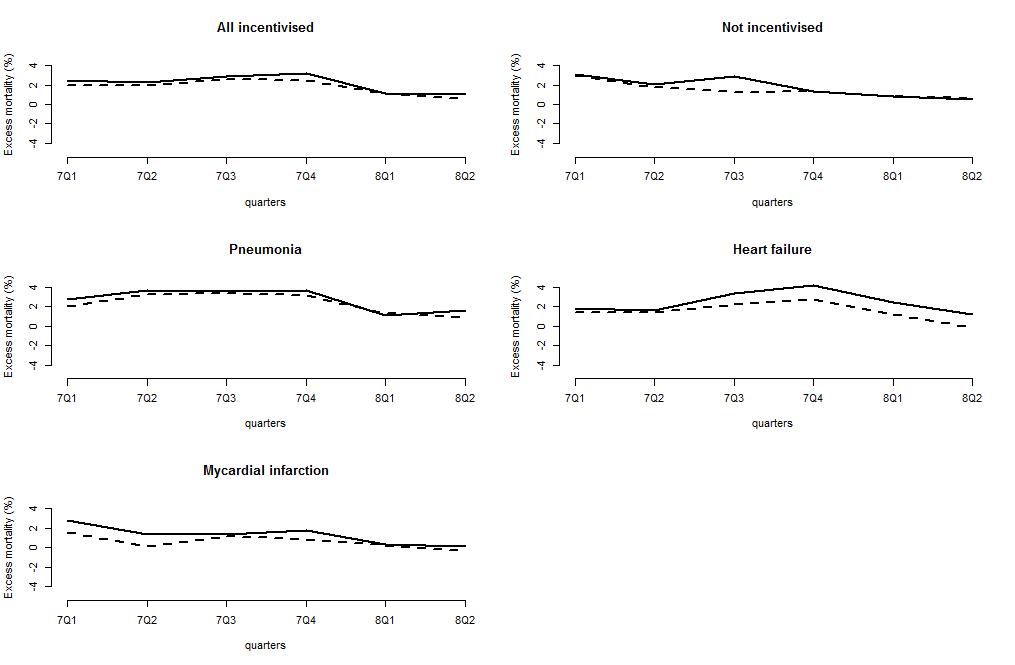


**Figure 1 Pre-treatment trajectories of risk-adjusted mortality, after multivariate matching. Solid line: North West. Dashed line: matched hospitals of the rest of England.**

**Appendix 7: Assessing the sensitivity of the synthetic control analysis to constraining the donor pool**

| 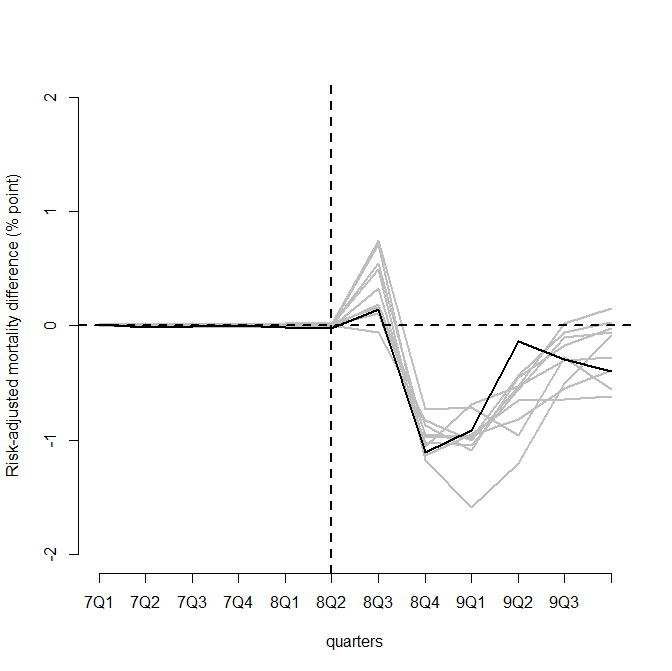 | 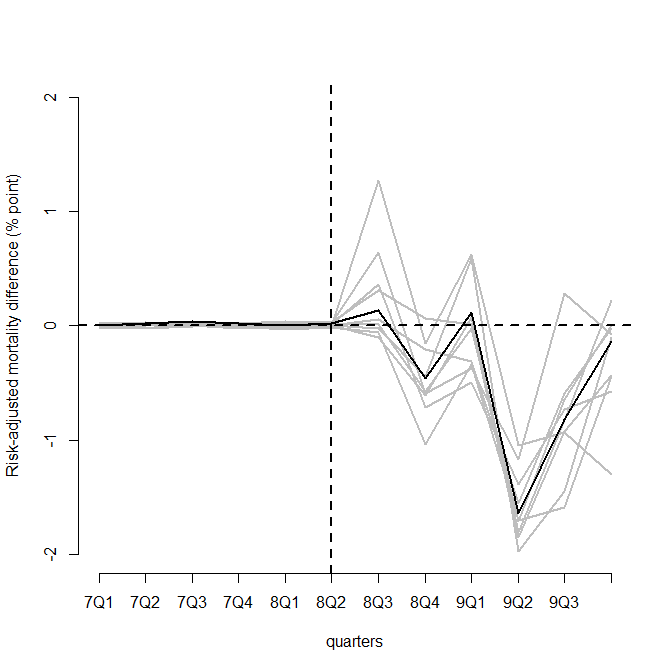 |
| --- | --- |

**Figure 1 The estimated gaps for the analysis with full sample (black line), and when leaving out all hospitals from each of the 9 control regions (grey lines). Left panel: all incentivised conditions. Right panel: pneumonia**
